# Ethanol Extract of Fructus Schisandrae Decreases Hepatic Triglyceride Level in Mice Fed with a High Fat/Cholesterol Diet, with Attention to Acute Toxicity

**DOI:** 10.1093/ecam/nep070

**Published:** 2011-01-04

**Authors:** Si-Yuan Pan, Zhi-Ling Yu, Hang Dong, Chun-Jing Xiang, Wang-Fun Fong, Kam-Ming Ko

**Affiliations:** ^1^Department of Pharmacology, Beijing University of Chinese Medicine, Beijing 100102, China; ^2^School of Chinese Medicine, Hong Kong Baptist University, Hong Kong; ^3^Department of Biochemistry, Hong Kong University of Science & Technology, Hong Kong

## Abstract

Effects of the ethanol extract of Fructus Schisandrae (EtFSC) on serum and liver lipid contents were investigated in mice fed with high fat/cholesterol (HFC) diet for 8 or 15 days. The induction of hypercholesterolemia by HFC diet caused significant increases in serum and hepatic total cholesterol (TC) levels (up to 62% and 165%, resp.) and hepatic triglyceride (TG) levels (up to 528%) in mice. EtFSC treatment (1 or 5 g/kg/day for 7 days; from Day 1 to 7 or from Day 8 to 14, i.g.) significantly decreased the hepatic TG level (down to 35%) and slightly increased the hepatic index (by 8%) in hypercholesterolemic mice. Whereas fenofibrate treatment (0.1 g/kg/day for 7 days, i.g.) significantly lowered the hepatic TG level (by 61%), it elevated the hepatic index (by 77%) in hypercholesterolemic mice. Acute toxicity test showed that EtFSC was relatively non-toxic, with an LD_50_ value of 35.63 ± 6.46 g/kg in mice. The results indicate that EtFSC treatment can invariably decrease hepatic TG in hypercholesterolemic mice, as assessed by both preventive and therapeutic protocols, suggesting its potential use for fatty liver treatment.

## 1. Introduction

Fatty liver, characterized by the buildup of fat in liver cells, is prevalent in modern societies. In USA as well as European and Asian countries, the incidence of fatty liver is 15–20% of the general population, and it is much higher in obese individuals [1–4]. Although fat present in the liver usually causes no damage by itself, in some situations abnormal accumulation of lipids in the liver may interfere with normal liver functions and causes hepatocyte injury, hepatocirrhosis, and hepatocellular carcinoma [5–7]. It is therefore of great pharmacological interest to develop drugs that may prevent or ameliorate liver steatosis. Fatty liver is also associated with diabetes mellitus, tuberculosis, malnutrition, excess vitamin A in the body, or the uses of certain drugs, and it occurs even as a complication of pregnancy [8]. Severe fatty liver is more often due to improper diet, alcohol excess and obesity. At present, fatty liver is a commonly occurring health problem.

Fructus Schisandrae (FSC), the fruit of *Schisandra chinensis*, is a traditional Chinese herb (*Wu-Wei-Zi*) originally recorded in *Shen Nong Ben Cao Jing* (an over 2000 years old Herbal Pharmacopoeia in China). Its active components and related synthetic derivatives, such as schisandrin B, bifendate and bicyclol, have been shown to reduce the hepatotoxicity produced by chemicals in animals and to treat patients with chronic viral hepatitis in clinic conditions [9–14]. The hepatic lipid-lowering effect of bicyclol treatment on alcohol-induced fatty liver has also been demonstrated in mice [15]. Our previous studies have shown that the treatment with bifendate, bicyclol or schisandrin B antagonized hepatic steatosis in mice with hypercholesterolemia caused by feeding a cholesterol/bile salt or high-fat diet [16–20]. In the present study, we endeavored to investigate the effects of the ethanol extract of FSC (EtFSC) on serum/hepatic triglyceride (TG) and total cholesterol (TC) levels in hypercholesterolemic mice produced by high fat/cholesterol (HFC) diet, with an objective of establishing a pharmacological basis for its potential application in the treatment of fatty liver.

## 2. Methods

### 2.1. Preparation of Herbal Extract

FSC was obtained from the Anguo Chinese *material medica* market and authenticated by Dr H. Dong. FSC was ground into small pieces and extracted twice with five volumes of 80% (v/v, in H_2_O) ethanol under reflux. The extracts were combined and filtered through a paper filter. The pooled extract was concentrated by rota-evaporation under reduced pressure until the removal of all ethanol. EtFSC was reconstituted in water (with 2 g of raw herb in 1 ml of extract) and stored at 4°C until use.

### 2.2. Chemicals and Reagents

Cholesterol (certificate no. 041103) was obtained from Beijing Chemical Reagent Co. (Beijing, China). Fenofibrate (certificate no. 0405030) was bought from Beijing Yongkang Medical Ltd. (Beijing, China). Sodium carboxymethylcellulose (CMC, certificate no. 971230) was obtained from Beijing Xudong Chemical Plant (Beijing, China). Assay kits for TC and TG were bought from Zhongsheng Beikong Bio-technology and Science Inc. (Beijing, China).

### 2.3. Animal Treatment

Male ICR mice (Grade II, certificate No. SCXK(jing) 2002–0003), weighing 18–20 g, were supplied by Vital River Lab Animal Co. Ltd (Beijing, China). All animals were maintained on a 12 h (light on 7:00–19:00 h) light-dark cycle at 20-21°C, with a relative humidity of 50–55%. They were allowed free access to water and food. Experiments were performed when the animals had grown up to a body weight of 24–26 g. Hypercholesterolemia was induced by feeding high-fat diet (10% lard, w/w) containing cholesterol (1%, w/w). Non-hypercholesterolemic (normal) animals were given the normal diet. In the drug treatment groups, EtFSC or fenofibrate (suspended in 0.5% CMC) was intragastrically administered at doses of 1–5 (based on raw herb) or 0.1 g/kg, respectively. The dosage of EtFSC adopted for the present pharmacological investigation was much higher than the recommended intake of FSC (1.5–5 g daily) for a human adult with an average body weight of 60 kg.

The experimental design aimed to investigate the preventive and therapeutic effects of EtFSC treatment on liver steatosis. Mice were fed with HFC diet for 8 or 15 days. EtFSC (1–5 g/kg/day) or fenofibrate (0.1 g/kg/day) treatment was performed for 7 days (from Day 1 to 7 (preventive) or from Day 8 to 14 (therapeutic)). Drug-untreated animals received the vehicle (0.5% CMC) at 10 ml/kg. Animals were sacrificed 24 h after the last dosing with EtFSC or fenofibrate. Blood and liver tissue samples were obtained from ether-anesthetized animals which had been fasted for 6 h (from 6:00 to 12:00 h), and they were subjected to biochemical analyses. Hepatic index was estimated from the ratio of total liver weight to body weight. Experimental protocols were approved by the University Committee on Research Practice in Beijing University of Chinese Medicine.

### 2.4. Determination of Total Cholesterol and Triglyceride Levels

Serum samples were prepared by centrifuging the whole blood obtained from the orbital vein for 8 min at 2000 × g. The liver tissue sample was homogenized in 9 volumes of 0.9% (w/v) NaCl solution by two 10-s bursts of a tissue disintegrator and the homogenate was then centrifuged at 2000 g for 15 min to obtain the supernatants. Aliquots of 10 and 40 *μ*l of the hepatic supernatants were used to determine the TG and TC levels, respectively.

### 2.5. Statistical Analysis

Data were analyzed using Student's *t*-test and *P* < .05 was considered significant. Values are expressed as means ± SEM for 10 mice per group.

## 3. Results

### 3.1. EtFSC/Fenofibrate Treatment Changed Serum Triglyceride and Total Cholesterol Level in Hypercholesterolemic Mice

**Figure 1:**
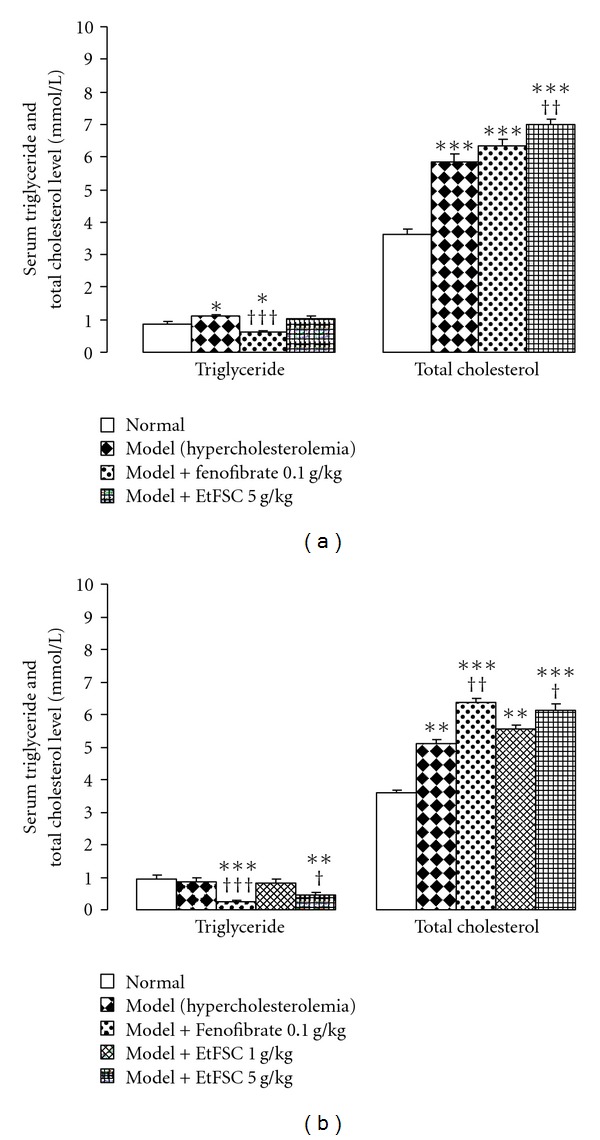
Effects of EtFSC/fenofibrate treatment on serum triglyceride and total cholesterol levels in mice fed with HFC for 8 days (a) or 15 days (b). EtFSC (1–5 g/kg/day, i.g.) or fenofibrate (0.1 g/kg/day, i.g.) treatment was performed for 7 days (from Day 1 to 7 or from Day 8 to 14). Normal and drug-untreated hypercholesterolemic animals received the vehicle (0.5% CMC 10 ml/kg/day, i.g.) only. Twenty-four hours after the last dosing, serum triglyceride and total cholesterol levels were determined. Values given are the mean ± SEM of 10 mice per group. *P < .05, **P < .01 and ***P < .001 versus the normal group; ^†^ *P* < .05, ^††^ *P* < .01 and ^†††^ *P* < .001 versus the drug-untreated hypercholesterolemic group.

Feeding mice with HFC diet for 8 days significantly increased serum TC and TG levels (by 62% and 29%, resp.), when compared with animals fed with normal diet (Figure 1(a)). EtFSC and fenofibrate treatment (from Day 1 to 7) increased serum TC levels (by 8% and 20%, resp.), when compared with the drug-untreated hypercholesterolemic mice. The fenofibrate, but not EtFSC, treatment significantly reduced serum TG levels (by 45%) in hypercholesterolemic mice (Figure 1(a)).

As shown in Figure 1(b), feeding mice with HFC diet for 15 days increased the serum TC levels (by 50%), but it did not affect the serum TG level. Both EtFSC and fenofibrate treatments (from Day 8 to 14) increased the serum TC level (by 2–19%). In contrast, they decreased the serum TG level (by 44% and 72%, resp.) in hypercholesterolemic mice.

### 3.2. EtFSC/Fenofibrate Treatment Decreased Hepatic Triglyceride and/or Total Cholesterol Level in Hypercholesterolemic Mice

**Figure 2:**
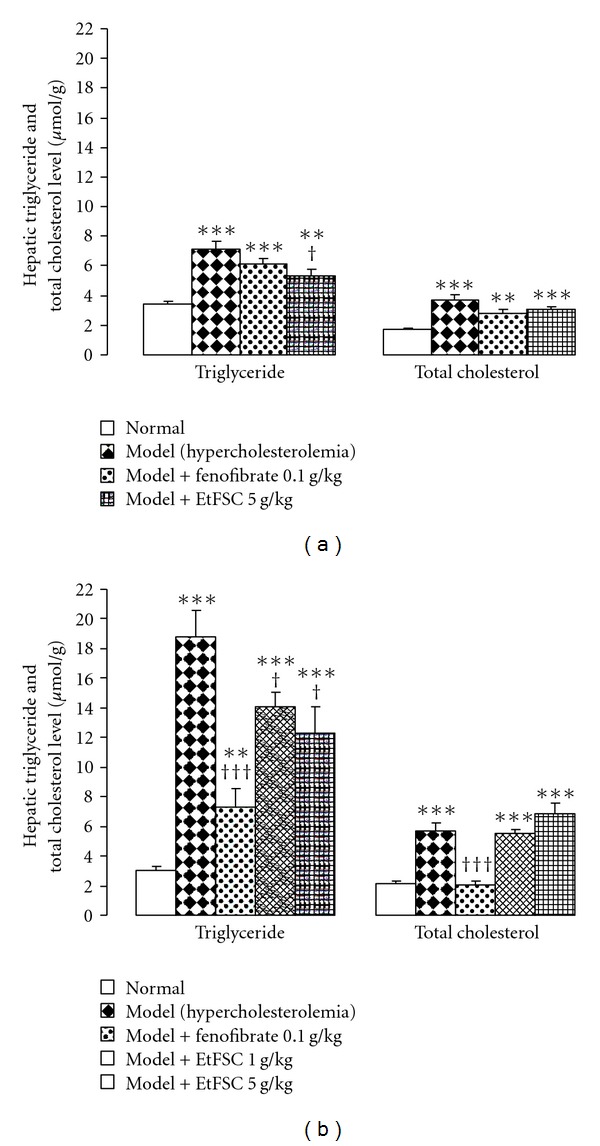
Effects of EtFSC/fenofibrate treatment on hepatic triglyceride and total cholesterol levels in mice fed with HFC for 8 days (a) or 15 days (b). Experimental details were described in Figure 1. Twenty-four hours after the last dosing, hepatic triglyceride and total cholesterol levels were determined. Values given are the mean ± SEM of 10 mice per group. **P < .01 and ***P < .001 versus the normal group; ^†^ *P* < .05 and ^†††^ *P* < .001 versus the drug-untreated hypercholesterolemic group.

Feeding mice with HFC diet for 8 days significantly increased hepatic TC and TG levels (by 118% and 107%, resp.), when compared with animals fed with normal diet. EtFSC treatment (5 g/kg/day × 7, from Day 1 to 7) significantly decreased hepatic TG levels (by 26%), when compared with the drug-untreated hypercholesterolemic mice. Hepatic TC level was not affected by EtFSC or fenofibrate treatment in mice fed with HFC diet (Figure 2(a)).

Feeding mice with HFC diet for 15 days increased hepatic TC (by 165%) and TG (by 528%) levels, when compared with that of mice fed with normal diet. EtFSC treatment (1 or 5 g/kg/day × 7, from Day 8 to 14) produced dose-dependent decreases in hepatic TG levels (by 25–35%), when compared with the drug-untreated hypercholesterolemic mice. Fenofibrate significantly decreased both hepatic TG and TC levels (by 61% and 64%, resp.), when compared with the drug-untreated hypercholesterolemic mice (Figure 2(b)).

### 3.3. Effects of EtFSC/Fenofibrate Treatment on Liver Weight in Hypercholesterolemic Mice

**Figure 3:**
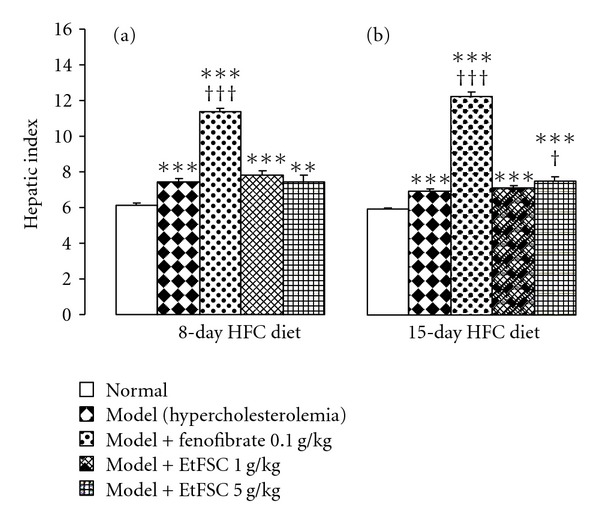
Effects of EtFSC/fenofibrate treatment on hepatic index in hypercholesterolemic mice. Experimental details for insets (a) and (b) were described in Figure 1. Hepatic index was estimated by the ratio of the whole liver weight to body weight. Values given are the mean ± SEM of 10 mice per group. **P < .01 and ***P < .001 versus the normal group; ^†^ *P* < .05 and ^†††^ *P* < .001 versus the drug-untreated hypercholesterolemic group.

Figure 3 shows the EtFSC/fenofibrate-induced changes in hepatic index in HFC diet-fed mice. The hepatic index was increased in hypercolesterolemic mice (up to 21%), when compared with the normal diet-fed mice. EtFSC treatment increased the hepatic index (by 8%) in 15-day HFC diet-fed mice (Figure 3b), but it did not affect the hepatic index in 8-day HFC diet-fed mice (Figure 3a). Fenofibrate significantly elevated the hepatic index (up to 77%) in both 8- and 15-day HFC diet-fed mice, when compared with the drug-untreated hypercholesterolemic mice (Figures 3a and 3b).

### 3.4. Acute Toxicity of EtFSC

In the acute toxicity test, EtFSC was administered intragastrically at increasing doses (19.6–57 g/kg) and the mortality rate was determined within 24 h post-dosing in mice. LD_50_ value of EtFSC was estimated to be 35.63 ± 6.46 g/kg, using the Bliss method (Table 1).

## 4. Discussion

Fatty liver is caused by sedentary lifestyle, diet enriched with cholesterol and saturated fats, and medical conditions. Lifestyle contributors include obesity, deprivation of physical exercise, and excessive alcohol consumption [21]. In the present study, feeding with HFC diet for 8 or 15 days markedly increased serum TC, hepatic TG and TC levels in mice, with the extent of elevation for hepatic TG being more prominent. Serum TG level was slightly increased in mice fed with HFC diet for 8 but not 15 days. The animal model used in the present study therefore mimics the clinical condition of fatty liver rather than hyperlipidemia. In most instances, the treatment of fatty liver requires a proper control of the underlying abnormal conditions, including high blood TG level, diabetes, excessive alcohol intake and obesity [22]. At present, orthodox medicine has no specific drug treatment to reverse a condition of fatty liver. Moreover, most of the synthetic lipid-lowering drugs can lead to various adverse reactions, even though they can alleviate the condition of hepatic steatosis. As such, the World Health Organization has adopted a policy in favor for development of herbal remedies for the clinical management of hepatic steatosis.

The acute treatment with EtFSC increased the serum TG level and at the same time, decreased hepatic TC level in normal mice. As expected, the hypertriglyceridemic action of EtFSC is suppressed by the co-administration of fenofibrate (data not shown). Fenofibrate, a broad-spectrum lipid-lowering drug of the fibrate class, inhibits the synthesis of cholesterol and triglycerides as well as enhances their elimination [23, 24]. In the present study, it was found that both fenofibrate and EtFSC treatment for 7 days reduced the serum TG level but elevated the serum TC levels in HFC diet-induced hypercholesterolemic mice. The blood TG lowering effect may be related to the high blood TC levels in HFC diet-fed mice. Possibly, hypercholesterolemia may facilitate the utilization of or inhibit the synthesis of TG. The fenofibrate- or EtFSC-induced increase in serum TC levels may result from the secretion of hepatic TC into the blood stream. While fenofibrate did not reduce hepatic TC levels in mice fed with HFC diet for 8 days, it lowered hepatic TC in mice fed with HFC diet for 15 days. This observation may be due to a higher content of hepatic TC in mice fed with HFC diet for 15 days than those fed with HFC diet for 8 days.

It has been reported that the hypolipidemic action of fenofibrate is attributed to the stimulation of lipolysis and the elimination of TG-rich particles from plasma through activating lipoprotein lipase and reducing the production of apolipoprotein C-III, as well as inducing enzymes for catalyzing *β*-oxidation of fatty acid in mitochondria [25–27]. In addition, fenofibrate also reduces intestinal cholesterol absorption [28]. Therefore, the mechanism involved in the reduction of hepatic TG level in hypercholesterolemic mice by EtFSC treatment may also be due to its effect on the lipoprotein lipase, apolipoprotein C-III, *β*-oxidation, or/and cholesterol absorption. The detail mechanism of action remains to be elucidated. In our previous study, the treatment of schisandrin B, bifendate (synthetic intermediate of schisandrin C) and bicyclol (synthetic dibenzocyclooctadiene derivative) [16–20] produced a significant hepatotrophic effect, but EtFSC treatment only slightly increased the hepatic index in hypercholesterolemic mice. The lesser degree of liver hypertrophy caused by EtFSC than purified active principles including schisandrin B, bifendate, and bicyclol and fenofibrate, and the relatively low toxicity might make EtFSC a desirable candidate herbal preparation for the treatment of fatty liver.

**Figure 4:**
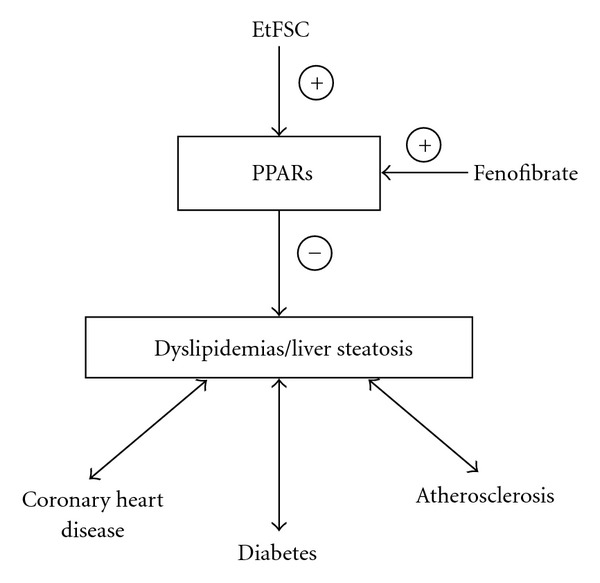
Hypothetical action of EtFSC in lowering liver lipid content through the intermedicacy of PPARs.

Peroxisome proliferator-activated receptors (PPARs) play important roles in the regulation of lipid metabolism involved in inflammation, energy balance, atherosclerosis and non-alcoholic fatty liver disease [29–31]. Fenofibrate, an agonist of PPAR*α*, is a useful therapeutic option for patients with dyslipidemias, particularly those associated with diabetes mellitus or coronary heart disease, and metabolic syndrome [32, 33]. EtFSC may activate PPARs and thereby suppress the lipid accumulation in the liver (Figure 4).

In conclusion, EtFSC treatment could prevent or ameliorate the degree of liver steatosis in hypercholesterolemic mice. While fenofibrate produced a more potent lipid-lowering action than that of EtFSC, it caused a larger extent of liver hypertrophy. Taken together with the relatively low toxicity, EtFSC may be used clinically for the management of fatty liver disease [34].

## Funding

Natural Science Foundation of Beijing City (Grant no. 7022017); Hong Kong Baptist University (Grant no. FGR/06-07/II-67).

## Figures and Tables

**Table 1:** Acute toxicity of EtFSC in mice.

Dose (g/kg)	Number of animal	Mortality rate (%)
19.6	10	10
28.0	10	30
40.0	10	40
57.0	10	100

Mice were divided into four groups of 10 animals in each and orally administered with EtFSC at doses of 19.6–57 g/kg. The mortality rate in each group was determined within 24-h post-dosing. LD_50_ value (35.63 ± 6.46 g/kg) was estimated by Bliss method.
